# Diffusion weighted imaging and blood oxygen level-dependent MR imaging of kidneys in patients with lupus nephritis

**DOI:** 10.1186/s12967-014-0295-x

**Published:** 2014-10-24

**Authors:** Xiao Li, Xueqin Xu, Qianying Zhang, Hong Ren, Wen Zhang, Yan Liu, Fuhua Yan, Nan Chen

**Affiliations:** Department of Nephrology, Ruijin Hospital, Shanghai Jiao Tong University School of Medicine, Shanghai, China; Department of Radiology, Ruijin Hospital, Shanghai Jiao Tong University School of Medicine, Shanghai, China

**Keywords:** Lupus nephritis, Functional MR imaging, Renal function, Pathological changes

## Abstract

**Background:**

Lupus nephritis (LN) is one of most common secondary glomerulonephritis. There is no ideal method to simultaneously assess renal structure and function in patients with LN. The aim of this study is to investigate the utility of diffusion weighted imaging (DWI) and blood oxygen level-dependent (BOLD) MR imaging in the assessment of renal involvement and pathological changes in patients with LN.

**Methods:**

Sixty-five patients with LN and 16 healthy volunteers underwent coronal echo-planar DWI and BOLD MR imaging of the kidneys. The apparent diffusion coefficient (ADC) and R2* values of the kidneys were calculated with *b* values of 0 and 500 s/mm^2^. The relationship between the renal injury variables and the ADCs or R2* values were evaluated. And 16 of 65 patients with LN underwent a repeated evaluation after the induction treatment for 9 to 12 months.

**Results:**

The mean ADC values of kidneys in patients with LN were 2.40 ± 0.25 × 10^−3^ mm^2^/ s, the mean R2* values of the renal cortex and medulla were 11.03 ± 1.60/sec and 14.05 ± 3.38/sec respectively, which were all significantly lower than that in volunteers. In patients with LN, the mean ADC values were correlated with eGFR (r = 0.510, p < 0.01). There was a negative correlation between the mean ADC values and renal pathology chronicity indexes (r = −0.249, p < 0.05), the R2* values of the renal medulla and proteinuria (r = −0.244, p < 0.05), and the degree of tubulointerstitial lesions (r = −0.242, p < 0.05). The ADC and R2* values of kidneys were significantly higher than those of pre-treatment in complete remission patients.

**Conclusions:**

DWI and BOLD MR imaging of kidneys may be used to noninvasively monitor the disease activity and evaluate therapeutic efficacy in lupus nephritis.

## Background

Systemic lupus erythematosus (SLE) is an autoimmune disease with variable presentations, course and prognosis. Approximately half a million people in Europe and seventy per 100 000 people in China suffer from SLE. Renal involvement is one of the major determinants of the outcome in patients with SLE [1]. Lupus nephritis (LN) is one of the most common secondary glomerulonephritis in China. Renal interstitial infiltrates, tubular necrosis and interstitial fibrosis can be found in 60-70% patients with LN. Interstitial lesions are often associated with glomerular function, and may play a pivotal role on progression and prognosis of lupus nephritis [2-4]. Nowadays there is no ideal method to simultaneously assess renal structure and function in patients with LN. Moreover, effective means to evaluate tubulointerstitial hypoxia state is lack as well. Therefore, it’s difficult to dynamically monitor the disease progression in these patients.

Functional magnetic resonance (MR) imaging techniques such as diffusion-weighted imaging (DWI) and blood oxygen level-dependent (BOLD) imaging have shown considerable value in the evaluation of renal function in health and renal diseases [5-7]. The aim of this study is to investigate the feasibility of functional renal MR imaging in the assessment of renal functional and structural changes in patients with lupus nephritis.

## Methods

### Study population

Sixty-five patients with lupus nephritis, diagnosed according to clinical manifestation, laboratory findings and renal histopathological changes, had been followed up since their admission in our department during the recent two years. They all met the diagnosis criteria of systemic lupus erythematosus defined by the American Rheumatism Association in 1997. All the patients were included after signing informed consent. There were 10 male and 55 female patients, with mean age of 34 ± 12 years old. Sixteen healthy volunteers, 2 men and 14 women with mean age of 35 ± 11 years old, had no history of primary or secondary renal diseases.

### MR imaging

All the LN patients and healthy volunteers underwent coronal echo-planar DW and BOLD MR imaging of the kidneys with a single breath-hold time. And 16 of the 65 patients with LN received functional MR imaging of kidneys again after 9 to 12 months induction treatment with immunosuppression. All of them fasted for 4–8 hours before the examination. MR imaging was performed with a 1.5 T MR imager (GE Twin-Speed, General Electric Medical Systems, Milwaukee, WI). DW MR imaging was performed with a spin echo-echo planar imaging (SE-EPI) sequence, and BOLD MRI was performed with a multiple gradient-recalled-echo (mGRE) sequence. The following parameters were used for DWI: 11 sections (section thickness, 5 mm; intersection gap, 1 mm); repetition time (TR), 2000 ms; echo time (TE), 52.9 ms; NEX, 2; field of view, 380 × 380 mm; matrix, 128 × 128; acquisition time, 16 s. The diffusion gradient b values of 0 and 500 s/mm^2^ were used. The following parameters were used for BOLD MRI: TR/TE, 110 ms/2.2-27.5 ms; flip angle, 60°; matrix, 132x128; section thickness, 5 mm; intersection gap, 1 mm. Three coronal sections were obtained for each kidney and eight T2*-weighted images were acquired for each section within one 16-second breath hold.

### Imaging analysis

The apparent diffusion coefficient (ADC) and R2* values of the kidneys were calculated on a GE workstation (Sun Microsystems, ADW4.2) with Functool 2 image analysis software. In the transverse and coronal ADC map, freehand regions of interest (ROIs) were placed on the parenchyma of the kidneys, and fat within renal sinus were excluded. Three such ROIs were placed—one each in the upper pole, inter-polar region, and lower pole—and the mean of these three values were calculated.

R2* values of renal cortex and medulla were measured on T2* maps. The size of ROI was set to reduce the noise and the influence of partial volume effects. Avoiding visible vessels, ROIs were placed manually on the clear corticomedullary differentiation image. Three ROIs were placed on each side of renal cortex and medulla, and the mean of R2* values were calculated.

The relationship between the renal injury variables and the ADCs or R2* values were evaluated. The renal function was estimated using the abbreviated modification of diet in renal disease (MDRD) study equation. Renal tubulointerstitial lesions were scored with semi quantitative method: no lesion was 0, mild lesion (≤25%) was 1, moderate lesion (25% ~ 50%) was 2, and severe lesion (≥50%) was 3. There were 0–9 scores for tubulointerstitial lesions including 0–3 for tubular atrophy, 0–3 for interstitial fibrosis, 0–3 for interstitial inflammatory cells infiltration. The renal pathology activity index (AI) and chronicity index (CI) were evaluated in patients with LN.

### Statistical analysis

The SPSS software was used (version 13.0; SPSS; Chicago, Illinois, USA). All results were expressed as mean ± SD for normally distributed data, median and range for skewed data and frequency (%) for categorical data. The distribution of clinical and laboratory attributes among the groups were evaluated by *t* test. The Pearson correlation analysis was used to evaluate the relation between the clinical or pathological variables and the ADCs or R2* values. *P* < 0.05 was considered as statistically significant.

## Results

### The mean ADC and R2* values of kidneys in patients with LN were lower than that in healthy volunteers

**Figure 1:**
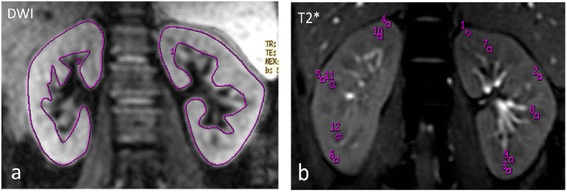
**Renal functional MR imaging of Patient A with LN-IV(S)A. (a)** DWI imaging. **(b)** T2* imaging. The proteinuria was 1.3 g/24 h and serum creatinine was 52 μmol/L, the renal pathology AI was 12 and CI was 0. Renal BOLD images showed a clear demarcation between cortex and medulla. The ADC value of left kidney was 2.81 x10^−3^ mm^2^/s and that of right kidney was 2.78 x10^−3^ mm^2^/s, the R2* values were similar to normal kidneys.

**Figure 2:**
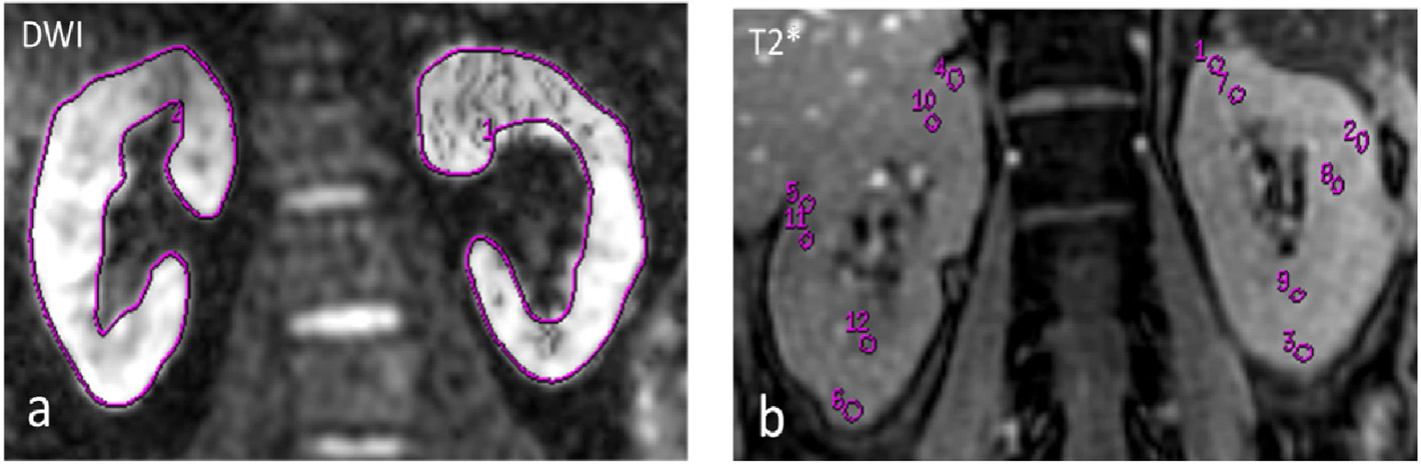
**Renal functional MR imaging of Patient B with LN-IV(G)A/C + V. (a)** DWI imaging. **(b)** T2* imaging. The proteinuria was 6.1 g/24 h and serum creatinine was 422 μmol/L, the renal pathology AI was 19 and CI was 4. The differentiation between cortex and medulla became less apparent in T2* imaging. The ADC value of left kidney was 2.14 x10^−3^ mm^2^/s and that of right kidney was 2.20 x10^−3^ mm^2^/s, the R2* values of cortex and medulla were 10.79/s and 8.67/s respectively.

The mean ADC values of kidneys in healthy volunteers were 2.52 ± 0.17 × 10^−3^ mm^2^/ s, the mean R2* values of the renal cortex and medulla were 12.63 ± 1.40/sec and 18.14 ± 2.51/sec respectively. The mean ADC values of kidneys in patients with LN were 2.40 ± 0.25 × 10^−3^ mm^2^/ s, the mean R2* values of the renal cortex and medulla were 11.03 ± 1.60/sec and 14.05 ± 3.38/sec respectively, which were all significantly lower than the ADC and R2* values of kidneys in healthy volunteers (p = 0.048, p = 0.045 and p = 0.008, respectively) (Table 1). There was no significant difference of the ADC and R2* values between bilateral kidneys either in volunteers or in patients with LN. Moreover, the ADC and R2* values were much lower in patients with severe lupus nephritis (Figures 1 and 2).

**Table 1:** The mean ADC and R2* values of kidneys in patients with LN compared with healthy volunteers $\left(\overline{\boldsymbol{x}} \pm \kern0.5em \boldsymbol{s}\right)$

	**ADC (×10** ^**−3**^ **mm** ^**2**^ **/ s)**	R2* of cortex (1/sec)	R2*of medulla (1/sec)
**Normal kidneys**	2.52 ± 0.17	12.63 ± 1.40	18.14 ± 2.51
**Kidneys with LN**	2.40 ± 0.25	11.03 ± 1.60	14.05 ± 3.38
**P value**	0.048	0.045	0.008

ADC: apparent diffusion coefficient; LN: lupus nephritis.

### The correlation between imaging values and renal function or pathological changes in patients with LN

**Figure 3:**
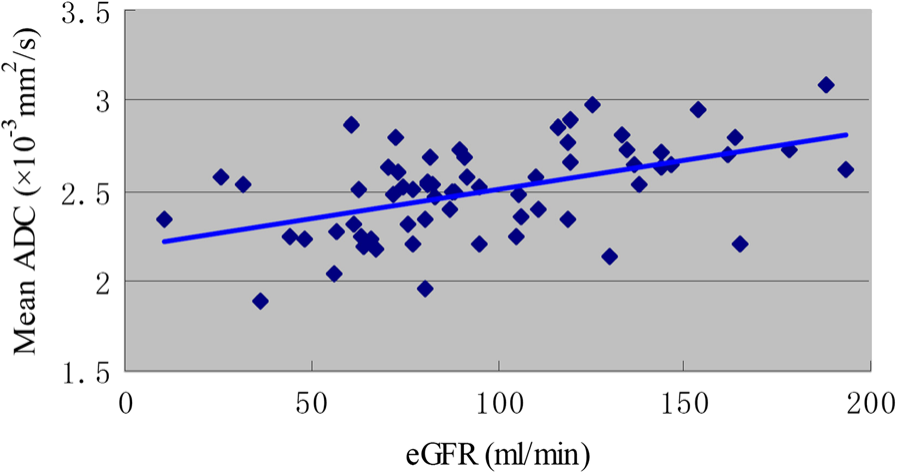
**The relationship of mean ADC and eGFR.** In the patients with LN, the mean ADC values were significantly correlated with eGFR (r = 0.510, p < 0.01).

In the patients with LN, the mean ADC values were correlated with glomerular filtration rate estimated by abbreviated MDRD equation (eGFR) (r = 0.510, p < 0.01) (Figure 3). There was a negative correlation between the mean ADC values and pathology chronicity indexes (r = −0.249, p < 0.05). Moreover, the R2* values of the renal medulla were negatively correlated with 24 hours proteinuria (r = −0.244, p < 0.05), the degree of tubulointerstitial lesions (r = −0.242, p < 0.05).

### Comparison of the mean ADC and R2* values of kidneys in two groups of LN

Of the 65 patients with LN, 6 were class I or II, 59 were class III, IV, V or combination. Fifty-nine patients were divided into 2 groups: Group A including 31 patients with LN class III, IV or V, and Group B including 28 patients with LN class V + III or V + IV. Urine N-acetyl-β-D-glucosaminidase (NAG) level in Group A was much lower than that in Group B (17.28 ± 11.23 *vs* 27.69 ± 13.24 U/L, p = 0.0037). There were 2 patients with abnormal tubular acidification, and 5 patients with impaired urinary concentrating function in Group A, while there being 9 patients with abnormal tubular acidification and 10 patients with impaired urinary concentrating function in Group B. The mean ADC values in Group A were higher than Group B ((2.50 ± 0.26) × 10^−3^ mm^2^/s *vs* (2.36 ± 0.21) × 10^−3^ mm^2^/s,p < 0.05). The mean R2* values of cortex in Group A were higher than Group B (11.70 ± 2.24/sec *vs* 10.57 ± 1.04/sec, p < 0.05), while mean R2* values of medulla in Group A being similar with that in Group B (13.99 ± 3.31/sec *vs* 13.78 ± 3.67/sec,p > 0.05) (Table 2).

**Table 2:** Comparison of the mean ADC and R2* values of kidneys in two groups of LN $\left(\overline{\boldsymbol{x}} \pm \kern0.5em \boldsymbol{s}\right)$

	**n**	**Mean ADC (×10** ^**−3**^ **mm** ^**2**^ **/ s)**	R2* values of cortex (1/sec)	R2* values of medulla (1/sec)
**Group A**	31	2.50 ± 0.26	11.70 ± 2.24	13.99 ± 3.31
**Group B**	28	2.36 ± 0.21^a^	10.57 ± 1.04^a^	13.78 ± 3.67

Compared with group A, ^a^p < 0.05.

Group A = class III, IV or V; Group B = class V + III or V + IV.

### The changes in ADC and R2* values of kidneys after the induction treatment in patients with LN

Among 16 patients who repeated MR scan after prednisone combined with immunosuppressant treatment in 9 to 12 months, 12 patients got complete remission (CR) and 4 had no response (NR). Complete remission was defined as urinary protein excretion that was less than 0.3 g/24 h, with normal urine sediment, and serum albumin concentration of greater than 35 g/L, and stabilization (±15%) or improvement in serum creatinine. The ADC and R2* values of kidneys were significantly higher than pre-treatment in CR patients (all p < 0.05), while the ADC and R2* values being lower than pre-treatment in NR patients with no statistical significance (p > 0.05) (Table 3).

**Table 3:** Comparison of the mean ADC and R2* values of kidneys in patients with LN before and after induction treatment

		**ADC (×10** ^**−3**^ **mm** ^**2**^ **/s)**	R2* of cortex (1/sec)	R2* of medulla (1/sec)
**Group CR**	**Before**	2.41 ± 0.16	10.25 ± 1.19	13.04 ± 3.15
	**After**	2.58 ± 0.14^a^	11.77 ± 1.11^a^	16.94 ± 3.50^a^
**Group NR**	**Before**	2.43 ± 0.12	10.84 ± 1.96	13.25 ± 0.41
	**After**	2.40 ± 0.23	10.13 ± 1.64	13.12 ± 0.47

^a^p < 0.05.

ADC: apparent diffusion coefficient; CR: complete remission; NR: no response.

## Discussion

The current evaluation of renal lesions in patients with lupus nephritis mainly depends on renal biopsy. Moreover, renal biopsy usually has to be repeated due to the possibility of changes in pathological patterns in LN. Although renal biopsy can provide the pathological information directly, it is not ideal for follow-up of treatment effects. Since it is invasive, SLE patients tend to have a great risk of bleeding due to coagulant function abnormality, or some patients are intolerable for renal biopsies if their kidneys are atrophy. Ultrasonography, commonly used in clinical practice, is simple and noninvasive. However, the accuracy of ultrasonic inspection is unstable depending on the skill of inspectors and body fat mass in patients. Scintigraphy is regarded as the gold standard for evaluating glomerular filtration rate (GFR), but the radioactive pollution, special equipment and other factors limit its clinical use scope [8]. Therefore, it seems critical to find a noninvasive and effective modality to dynamically evaluate renal function and pathological changes in patients with LN.

Because of its great promise in assessing physiological and pathophysiological parameters *in vivo*, functional magnetic resonance imaging has emerged as a useful technique to investigate organ functional characteristics beyond morphologic changes [9,10]. Kidneys are located at retro peritoneum and less affected by breath, with plenty of blood supply, high water content in tissues, and urine dilution and concentration function, that make kidneys become one of the most ideal organs for functional MR imaging [11,12]. DWI is a MR modality using strong bipolar gradients to create a sensitivity of the signal to the thermally induced Brownian motion of water molecules in tissue. Although the etiologies of chronic kidney diseases are different, their physiological and pathophysiological changes are usually common, including reduced blood supply of the kidneys, glomerulosclerosis, tubular atrophy and interstitial fibrosis, inducing abnormal diffusion of water molecules within renal tissue, which will contribute to ADC changes in diffusion-weighted imaging [13]. Diffusion of water molecules and perfusion of tissues can be affected by various renal lesions such as inflammation, necrosis, edema and fibrosis. The ADC (related only with water diffusion capacity) and perfusion scores (related to perfusion in the microvasculature) measured by DWI of multiple b-values can provide information concerning the characteristics of renal lesions and renal filtration function [14]. Chronic hypoxic injury in tubulointerstitium may be the common pathway of various chronic kidney diseases progressing to end stage renal disease (ESRD). BOLD MR is used for noninvasive assessment of the intrarenal oxygen content, based on paramagnetic properties of deoxyhemoglobin [12]. The change of R2* values can be found in the early stage of kidney damage in case of renal artery stenosis, unilateral ureteral obstruction, diabetes, hypertension and rejection after kidney transplantation [15,16].

Togao *et al*. [17,18] reported that DWI can reflect and monitor the progression of renal fibrosis in mice with unilateral ureteral obstruction, and ADC values were well associated with the changes in renal microstructure. It was found in our previous study [19] that DWI can be helpful to detect the early stage renal failure of CKD. We also found a linear correlation between the ADC values and the stages of CKD. Moreover, the ADC values reflected the unilateral renal function, while renal DWI was closely associated with renal tubulointerstitial lesions, especially tubular atrophy.

Karadeli *et al*. [20] found significant difference between the ADC_low_ values and the urine protein in patients with SLE, but no significant correlation between GFRs and ADCs. The limitation of their study was the small number of patients and mild SLE (only 10 cases with LN). To the best of our knowledge, our investigation is the first pilot study to evaluate and re-evaluate the renal functional and pathological changes before and after induction treatment in patients with LN by MR functional imaging. We found that renal ADC values and R2* values of cortex and medulla in LN were significantly lower than those in normal kidneys, and these values were significantly higher than before in complete remission patients (all p < 0.05). The results indicated that MR functional imaging had emerging as a new noninvasive method in the evaluation of renal prognosis and therapeutic effects.

There was no significant difference of the ADC values and R2* values of cortex or medulla between bilateral kidneys, reflecting the characteristics of diffuse lesions in LN. We analyzed the correlation of the mean ADC values of bilateral kidneys and eGFR which represents the overall filtration function of bilateral kidneys in patients with LN. It was found that the mean ADC values of kidneys were significantly correlated to eGFR in this study. The mechanism might be: Glomerular sclerosis, interstitial fibrosis and tubular atrophy leads to the decline in glomerular filtration rate, and the limitation in the intercellular movement of water molecules, which causing the abnormal diffusion of water molecules within renal tissues, and then the decrease in renal ADC values. It also can explain why there was a negative correlation between the mean ADC values and pathology chronicity indexes in LN.

Moreover, the R2* values of the renal medulla were negatively correlated with 24 hours proteinuria, the degree of tubulointerstitial lesions. In active lupus nephritis, glomerular cells proliferation or crescent formation, interstitial inflammatory cells infiltration, epithelial and endothelial immune complex deposition is common. Inflammatory factors and immune injury may lead to damage of microvasculature in glomeruli and surrounding renal tubules, which contribute to hemodynamic disturbance and hypoxia within kidneys. Chronic hypoxic injury may further induce glomerular sclerosis, interstitial fibrosis and tubular atrophy, which result in lower metabolism and reduction of oxygen consumption within kidneys, and finally cause a decrease in R2* values. Mixed membranous and proliferative LN (class V + III and V + IV) are usually known with more serious clinical presentation and more severe tubulointerstitial lesions than pure membranous LN (class V) or pure proliferative LN (class III and IV) [21]. In this study, R2* values of cortex in class V + III and V + IV were much lower than that in class III, IV or V, suggesting abnormal microcirculation and chronic hypoxia injury within kidneys with LN (V + III)/(V + IV). These findings should be considered preliminary.

## Conclusions

In summary, diffusion-weighted imaging and blood oxygen level-dependent MR imaging are novel functional MRI techniques with repeatable nature that have potential to assess glomerular filtration, parenchymal oxygenation and renal pathological changes. This pilot study indicated that DWI and BOLD MR imaging might be used to noninvasively monitor disease activity and evaluate therapeutic efficacy in lupus nephritis. Further studies involve larger group of patients with lupus nephritis are necessary.

## Abbreviations

LN: Lupus nephritis
DWI: Diffusion weighted imaging
BOLD: Blood oxygen level-dependent
MR: Magnetic resonance
SE-EPI: Spin echo-echo planar imaging
mGRE: Multiple gradient-recalled-echo
TR: Repetition time
TE: Echo time
ADC: Apparent diffusion coefficient
ROI: Regions of interest
MDRD: Modification of diet in renal disease
AI: Activity index
CI: Chronicity index
eGFR: Glomerular filtration rate estimated
NAG: N-acetyl-β-d-glucosaminidase
CR: Complete remission
NR: No response
ESRD: End stage renal disease
